# Selective activation of TNFR1 and NF-κB inhibition by a novel biyouyanagin analogue promotes apoptosis in acute leukemia cells

**DOI:** 10.1186/s12885-016-2310-5

**Published:** 2016-04-20

**Authors:** Christiana G. Savva, Sotirios Totokotsopoulos, Kyriakos C. Nicolaou, Christiana M. Neophytou, Andreas I. Constantinou

**Affiliations:** Department of Biological Sciences, University of Cyprus, Kallipoleos 75, Nicosia, 01678 Cyprus; Department of Chemistry, BioScience Research Collaborative, Rice University, 6500 Main Street, Houston, TX 77005 USA

**Keywords:** Biyouyanagin, Leukemia, Death receptors, Tumor necrosis factor receptor 1, TNFR1, Nuclear factor κΒ, NF-κΒ, Apoptosis, Caspases

## Abstract

**Background:**

Acquired resistance towards apoptosis is a hallmark of cancer. Elimination of cells bearing activated oncogenes or stimulation of tumor suppressor mediators may provide a selection pressure to overcome resistance. KC-53 is a novel biyouyanagin analogue known to elicit strong anti-inflammatory and anti-viral activity. The current study was designed to evaluate the anticancer efficacy and molecular mechanisms of KC-53 against human cancer cells.

**Methods:**

Using the MTT assay we examined initially how KC-53 affects the proliferation rates of thirteen representative human cancer cell lines in comparison to normal peripheral blood mononuclear cells (PBMCs) and immortalized cell lines. To decipher the key molecular events underlying its mode of action we selected the human promyelocytic leukemia HL-60 and the acute lymphocytic leukemia CCRF/CEM cell lines that were found to be the most sensitive to the antiproliferative effects of KC-53.

**Results:**

KC-53 promoted rapidly and irreversibly apoptosis in both leukemia cell lines at relatively low concentrations. Apoptosis was characterized by an increase in membrane-associated TNFR1, activation of Caspase-8 and proteolytic inactivation of the death domain kinase RIP1 indicating that KC-53 induced mainly the extrinsic/death receptor apoptotic pathway. Regardless, induction of the intrinsic/mitochondrial pathway was also achieved by Caspase-8 processing of Bid, activation of Caspase-9 and increased translocation of AIF to the nucleus. FADD protein knockdown restored HL-60 and CCRF/CEM cell viability and completely blocked KC-53-induced apoptosis. Furthermore, KC-53 administration dramatically inhibited TNFα-induced serine phosphorylation on TRAF2 and on IκBα hindering therefore p65/NF-κΒ translocation to nucleus. Reduced transcriptional expression of pro-inflammatory and pro-survival p65 target genes, confirmed that the agent functionally inhibited the transcriptional activity of p65.

**Conclusions:**

Our findings demonstrate, for the first time, the selective anticancer properties of KC-53 towards leukemic cell lines and provide a detailed understanding of the molecular events underlying its dual anti-proliferative and pro-apoptotic properties. These results provide new insights into the development of innovative and targeted therapies for the treatment of some forms of leukemia.

**Electronic supplementary material:**

The online version of this article (doi:10.1186/s12885-016-2310-5) contains supplementary material, which is available to authorized users.

## Background

Apoptosis deregulation occurs commonly in hematological malignancies and has been connected to cancer pathogenesis, progression and chemoresistance [1]. The two main effector cascades that are involved in apoptosis are the intrinsic (mitochondrial), and the extrinsic (death receptor) pathways [2]. Alterations affecting key molecules of these pathways such as Bcl-2, p53 and the nuclear factor κΒ (NF-κB) lead to accumulation of malignant cells. Among the latter, NF-κB promotes the transcription of genes encoding proteins involved in the suppression of cell death by both the intrinsic and the extrinsic pathway [3, 4]. Thus, elevation in NF-κB activity can increase cellular resistance to apoptosis.

The extrinsic pathway of apoptosis is initiated by engagement of cell surface receptors with specific ligands. The tumor necrosis factor receptor 1 (TNFR1) possesses important roles in many cellular responses. Once TNFR1 is stimulated by its ligand (TNFα), two complexes with opposing effects on cell fate can be formed: a pro-survival and a pro-apoptotic complex. In the presence of phosphorylated TNF receptor-associated factor 2 (TRAF2), pro-survival NF-κΒ activation dominates over pro-apoptotic Caspase-8 activation. TRAF2 phosphorylation, occurring on Ser 11, promotes receptor-interacting protein 1 (RIP1) ubiquitination, facilitating the recruitment and activation of the downstream IκB kinase complex (IKK). This leads to NF-κΒ activation [5, 6] and concurrently preventing RIP1 from interacting with Fas-associated death domain (FADD) protein and pro-caspase-8 [7, 8]. Under pro-apoptotic conditions, RIP1 dissociates from TRAF2 and binds to the FADD/Caspase-8 complex. Active Caspase-8 cleaves and inactivates RIP1 initiating the extrinsic pathway of apoptosis [9, 10].

Agents that trigger the extrinsic pathway are particularly intriguing, since nearly all anti-cancer drugs utilize the intrinsic pathway to induce apoptosis, and cells often become resistant by accumulating defects in this pathway (Reviewed in [11]). Consistent with this notion, deletions or mutations of *p53* [12, 13] or over-expression of *Bcl-2* [14, 15] and *NF-κB* [16] are common in acute myelocytic leukemia (AML) and acute lymphocytic leukemia (ALL) resulting in resistance to drugs that induce apoptosis through the intrinsic pathway. Consequently, the development of agents that trigger the extrinsic pathway of apoptosis is a promising approach for drug development against this disease [17–19].

Clinical trials aiming to evaluate the anticancer efficacy of TNF family members originated with the use of human TNFα mainly in advanced solid cancers [20, 21]. Recombinant human TNFα (rhTNFα) has been tested as a systemic treatment in several clinical trials and used as both a single agent and in combination with chemotherapeutics. Even though rhTNFα was proven as an effective anticancer agent in preclinical studies, these attempts were disappointing as clinical activity was rarely obtained; rhTNFα was unable to trigger apoptosis via TNFR1 unless the initial NF-κB pathway was blocked [22]. In addition, rhTNFα was highly cytotoxic towards hepatocytes causing severe side effects and lacked of evidence for therapeutic benefit [20]. Subsequently, for the development of rational death receptor-targeted therapy it is important to discover agents able to activate the death receptors without triggering the NF-κB cascade.

**Fig. 1:**
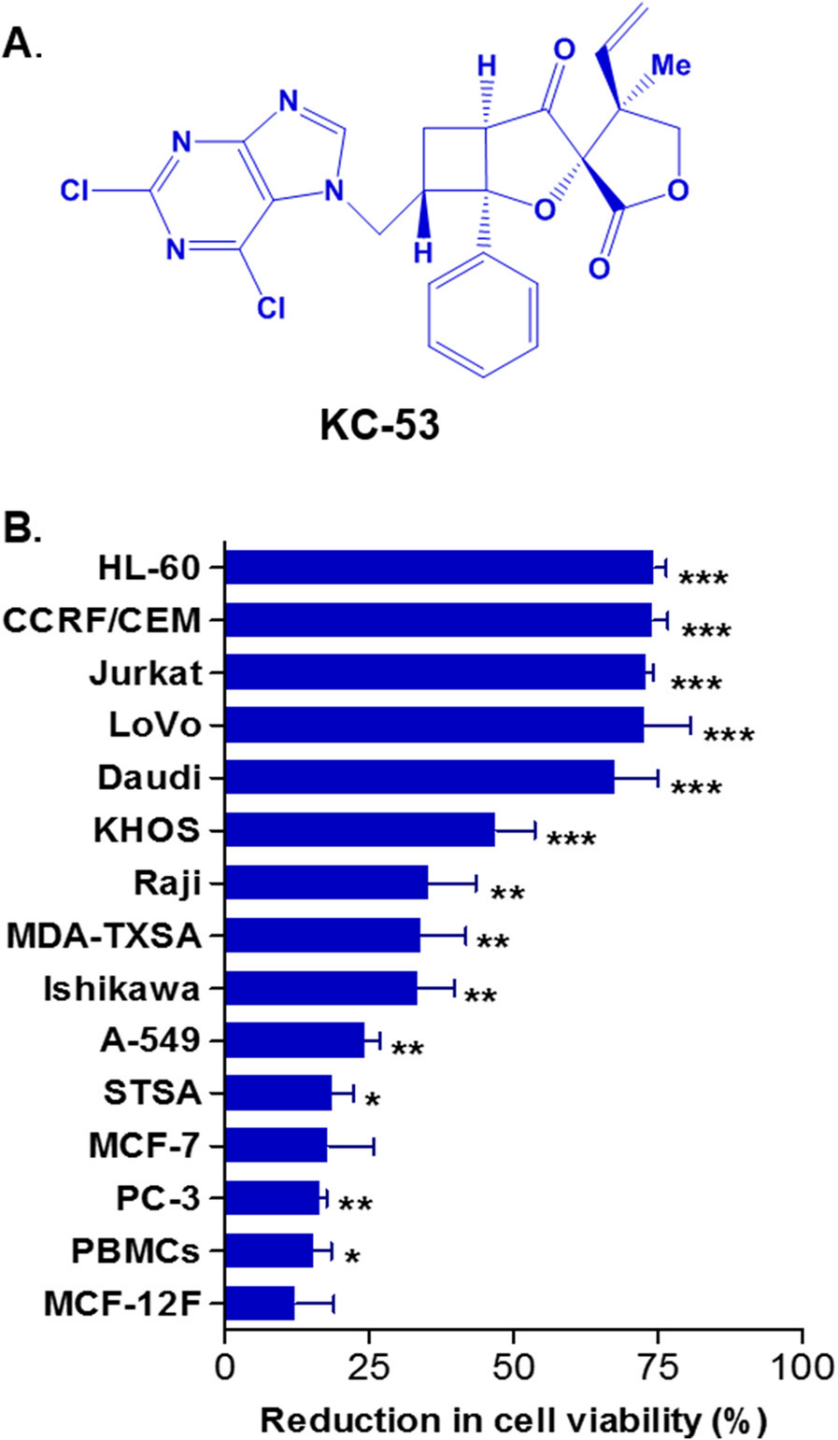
KC-53 chemical structure and its antiproliferative effects on a panel of cell lines and PBMCs. **a** Chemical structure of KC-53 molecule. **b** Cells were exposed to 5 μΜ of KC-53 for 48 h and cell survival was determined using the MTT assay. Cell viability is expressed as percentage of survival in vehicle treated cells. The results represent the mean ± SEM of three replicates and are representative of three different experiments. (*p value <0.05, **p value <0.01, ***p value <0.001)

Biyouyanagins are sesquiterpene spiro-lactones isolated from the plant *Hypericum chinense* with selective anti-virus and anti-inflammatory properties [23–26]. Our recent research around the molecular space of biyouyanagins structure revealed a new promising lead molecule; the post-photocycloaddition modified analogue 53 (Fig. 1a) [26]. Specifically, in THP-1 human macrophage cells, KC-53 inhibited the production and secretion of cytokines IL-6, IL-1β, and TNFα without affecting the production of cytokines IL-1α no 1β and IL-8 [26].

Since KC-53 was found to possess anti-inflammatory properties, and taking into consideration the key role of NF-κB in the inflammatory response, we postulated that, KC-53 may exhibit anticancer effects mediated through its interference with the TNFR1/NF-κB pathway. Our results show that among 13 cell lines tested, HL-60 (*p53*−/−) and CCRF/CEM (*p53*mut) are especially susceptible to the KC-53 pro-apoptotic effects due to, predominantly, activation of TNFR1 and the concomitant inhibition of p65/NF-κB translocation to the nucleus. The properties of KC-53, unveiled here, are consistent with those of a promising targeted therapeutic that could be especially effective in the treatment of some forms of leukemia that do not respond to drugs inducing only the intrinsic pathway of apoptosis.

## Methods

### Synthesis of KC-53

KC-53 was prepared by K.C. Nicolaou laboratory as previously described [26].

### Chemicals and reagents

FBS, Horse Serum, antibiotic/antimycotic, EGF, insulin, Cholera Toxin, Hydrocortizone, L-Glutamine, HEPES, Sodium Pyruvate and media used in cell culture were purchased from Gibco, Invitrogen (Carlsbad, California). Etoposide and Doxorubicin were purchased from Tocris (Bristol, UK). The pan caspase inhibitor z.vad.fmk was purchased from Sigma (St. Louis, Missouri). TNFα, and PS-341 (Bortezomib) were purchased from Merck Millipore (Darmstadt, Germany). Protease inhibitor cocktail was obtained from Roche (Indianapolis, IN). Caspase −3, −7, −8, −9, PARP1, RIP1, Bid, TNFR1, TNFR2, AIF, FADD, p-IκBα, p-TRAF2, p65/NF-κB and α-Tubulin antibodies were purchased from Cell Signaling Technology (Danvers, Massachusetts). Total TRAF2, total IκBα, Histone H3, EGFR, and GAPDH antibodies were obtained from Santa Cruz Biotechnology (Heidelberg, Germany). All other reagents were purchased from Sigma (St. Louis, Missouri).

### Cell culture

MCF-7, MDA-MB-231-TXSA, STSA, LoVo, Ishikawa and KHOS cells were cultured in DMEM supplemented with 10 % FBS, 1 % antibiotic/antimycotic and 4 mM L-Glutamine, PC-3, A-549, Jurkat, HL-60, CCRF/CEM, Raji and Daudi in RPMI supplemented with 10 % FBS, 1 % antibiotic/antimycotic and 4 mM L-Glutamine, and MCF-12F in DMEMF12 supplemented with 20 ng/mL EGF, 100 ng/mL Cholera Toxin, 500 ng/mL Hydrocortizone, 10 μg/mL insulin, 5 % Horse Serum and 1 % antibiotic/antimycotic. All cell lines were obtained from the American Type Culture Collection (ATCC) (Manassas, VA).

### Isolation of Peripheral Blood Mononuclear Cells (PBMCs)

Human normal PBMCs were isolated from heparinised venous blood samples by density gradient centrifugation method using Ficol-Histopaque (Sigma, St. Louis, Missouri). Briefly, the heparinised blood was layered on Histopaque in the ratio of 1:1 and subjected to centrifugation at 2,000 rpm for 30 min. The white layer representing PBMCs was aspirated out and transferred into sterile centrifuge tubes. The suspension of cells was then washed twice and cultured in RPMI supplemented with 10 % FBS, 1 % antibiotic/antimycotic and 4 mM L-Glutamine. After 24 h incubation at 37 °C non adherent cells (B- and T-cells) were collected for use in the experiments. Written informed consent was obtained from the donors of PBMCs and ethical approval was obtained from the Cyprus National Bioethics Committee in accordance with the Declaration of Helsinki.

### Proliferation assay

A total of 1 × 10^4^ cells were seeded per well of a 96-well plate in medium supplemented with the different concentrations of KC-53 or vehicle control, for the time periods described in the figure legends. At the end of each incubation period, MTT at a final concentration of 0.5 mg/mL was added to the medium and left to be metabolized for 3 h. Following that, plates were centrifuged at 1,500 rpm for 5 min. The medium was removed and DMSO was added in each well and incubated with gently shaking for 20 min. The absorbance measured at 570 nm, was proportional to the number of viable cells per well.

### Cell cycle analysis

Cells were added at a concentration of 1 × 10^6^ cells per a 100 mm plate and treated with KC-53 for indicated times at 37 °C. Following incubation, samples were harvested by centrifugation at 1,500 rpm for 5 min at 4 °C and washed with PBS. Cells were fixed with 70 % ethanol and stained with propidium iodide (PI) staining solution (0.2 mg/mL RNase A, 0.01 mg/mL PI). Samples were analyzed for DNA content using the Guava EasyCyte™ flow cytometer and the GuavaSoft analysis software (Millipore, Watford, UK).

### Annexin-V/PI staining

Cells were seeded at a concentration of 1 × 10^5^ cells per a 60 mm plate and treated with KC-53 or Doxorubicin (Dox) as indicated. Cells were harvested and stained using Annexin-V Alexa Fluor® 488/PI, as described by the Tali™ apoptosis kit (Life Technologies, Carlsbad, CA). Cell viability, death and apoptosis were evaluated using the Tali™ Image-based Cytometer (Life Technologies, Carlsbad, CA). The Annexin-V positive/PI negative cells were recognized as early apoptotic cells by the cytometer software whereas the Annexin-V positive/PI positive cells were identified as late apoptotic/dead cells.

### Cell death detection ELISA

Cells were added at a concentration of 1 × 10^4^ cells per well of a 96-well plate and treated with KC-53 in the present or absence of pan-caspase inhibitor, z.vad.fmk as indicated. The quantification of mono- and oligo-nucleosomes present in the cytoplasm of apoptotic cells was performed using the Cell Death ElisaPLUS Apoptosis Kit according to the manufacturer’s instructions (Roche, Indianapolis, IN). The specific Enrichment Factor of mono- and oligo-nucleosomes is expressed as absorbance of treated cells to absorbance of corresponding negative control.

### Caspase-8 enzymatic activity

Caspase-8 activity was measured using fluorogenic substrate IETD-AFC (KHZ0052) according to the manufacturer’s instructions (Invitrogen, California, USA). In brief, PBS-washed cell pellets were resuspended in Lysis buffer and incubated on ice for 10 min. Lysate was then centrifuged at 11,000 rpm for 5 min at 4 °C and supernatant-cytosolic fraction was collected. Substrate at 50 μΜ final concentration was added to 50 μg of cytosolic extract in each well of a 96-well plate followed by incubation for 1 h at 37 °C. Caspase activity was measured by monitoring the release of fluorigenic AFC using an auto-microplate reader (excitation 400 nm, emission 505 nm, slit width 15). Fold-increase in Caspase-8 activity was determined by direct comparison to the level of the uninduced control.

### Preparation of nuclear and cytosolic extracts

A total of 2 × 10^7^ cells were treated as indicated. After incubation, cells were harvested by centrifugation at 1,500 rpm for 5 min at 4 °C and washed with ice cold PBS. Cells were resuspended in ice cold Lysis buffer (10 mM HEPES, 1 mM EDTA, 60 mM KCl, 0.5 % (v/v) NP-40, 1 mM DTT, 1 mM PMSF, protease inhibitors, pH 7.9) and incubated at 4 °C for 10 min. Samples were then centrifuged at 12,000 g for 10 min at 4 °C and supernatant (cytosolic extract) was collected. Cytosolic fraction was further processed by centrifugation at 14,000 g, for 10 min at 4 °C. Supernatant was recollected and stored at −80 °C. Pellet was washed twice with Washing buffer (10 mM HEPES, 1 mM EDTA, 60 mM KCl, 1 mM DTT, 1 mM PMSF, protease inhibitors, pH 7.9) and Nuclear suspension buffer (250 mM Tris-Hydrochloride, 60 mM KCl, 1 mM DTT, 1 mM PMSF, protease inhibitors, pH 7.8) was added to each sample. Nucleus lysis was achieved by sonication (4 bursts, at amplitude 4, for 4 sec with 2 min cooling between bursts) with the use of an ultrasonic microprocessor and clarified by centrifugation at 10,000 g for 15 min at 4 °C. Supernatant (nuclear extract) was collected and stored at −80 °C.

### Quantitative real-time reverse transcription PCR (RT-qPCR)

Total RNA was extracted with Trizol reagent (Invitrogen, Carlsbad, CA) following the manufacturer’s protocol. cDNA was synthesized with random and oligo (dT) primers using the PrimeScript Reverse Transcriptase (TaKaRa Bio. Inc, Dalian, China). Primers were designed using Primer3 and are listed in Additional file 1. Real-Time PCR was performed using the BioRad CFX96 Real-Time System and the SYBR Green PCR Master Mix (Kapa Biosystems, Massachusetts) according to the manufacturer’s instructions. The PCR products were normalized to those obtained from human GAPDH mRNA amplification.

### RNA interference

FADD siRNA (sc-35352) and negative control siRNA (sc-37007) were purchased from Santa Cruz Biotechnology. For the transfection procedure, HL-60 and CCRF/CEM cells were seeded at a concentration of 4x10^5^/mL per well of a 12-well plate and FADD siRNA or control siRNA were transfected with the use of LipofetamineTM 2000 (Invitrogen, Carlsbad, CA) and siTransfection Reagent (Santa Cruz Biotechnology, Heidelberg, Germany) correspondingly according to the manufacturer’s instructions. The final concentration of siRNA in each well was 100 nM.

### Immunoblotting

Cells were treated as indicated and lysed with RIPA buffer (10 mM Tris-Cl (pH 8.0), 1 mM EDTA, 0.5 μΜ EGTA, 140 mM NaCl, 1 % Triton X-100, 0.1 % SDS, protease inhibior cocktail, phosphatase inhibitors; 5 mM NaF, 1 mM Na_3_VO_4_). For preparation of membrane and cytosolic extracts, the Subcellular Protein Fractionation Kit for Cultured Cells (PI-78840) was used according to the manufacturer’s instructions (Thermo Scientific, Rockford) with slight modifications. The total protein concentration was determined using Bradford reagent. Protein lysates were separated by electrophoresis on a 8–12 % SDS-PAGE gels and then electrophoretically transferred to PVDF membrane. Westerns blots were probed with the specific antibodies and protein bands were detected by enhcaned chemiluminescence. Anti-GAPDH, anti-Histone H3, anti-α-Tubulin and anti-EGFR monoclonal antibodies were used as loading controls. The intensity values from the densitometry analysis of Western blots were normalized against EGFR, α-Tubulin or Histone H3 using ImageJ analysis software (NIH). Intensity values were expressed as fold change compared to control.

### Statistical analysis

Results for continuous variables were presented as Mean ±Standard Error. Two- group differences in continuous variables were assessed by the unpaired T-test. *P*-values are two-tailed with confidence intervals 95 %. Statistical analysis was performed by comparing treated samples with vehicle controls. All statistical tests were conducted using Prism software version 5.0 (Graphpad, San Diego, California).

## Results

### KC-53 inhibits the proliferation of human cancer cell lines

The effect of KC-53 on tumor cell viability was initially determined in a series of human cancer cell lines to identify those that are the most sensitive to the agent. Thus, we have determined the effects of KC-53 on the viability of human breast (MCF-7, MDA-MB-231-TXSA), lung (A-549), prostate (PC-3), colon (LoVo), endometrial (Ishikawa), osteosarcoma (KHOS), gastric (STSA), leukemia (Jurkat, HL-60, CRF/CEM) and lymphoma (Raji, Daudi) tumorigenic cells. PBMCs and “normal” immortalized MCF-12 F breast cells were used as control cell lines. The HL-60 (AML/APL) and CCRF/CEM (ALL) cell lines were the most sensitive as determined by the IC_50_ at 48 h (Table 1). Importantly, the normal PBMCs and the immortalized MCF-12 F cells were relatively resistant to the anti-proliferative effects of the compound (Fig. 1b). KC-53 reduced cancer cell viability in a dose-depended manner in all cell lines with a maximum effect on the most sensitive cell lines ranging from 5 to 10 μM (Additional file 2). The two most sensitive cell lines were selected to further investigate the anti-proliferative mechanism of KC-53.

**Table 1:** IC_50_ values of KC-53 in vitro antiproliferative activity in human cell lines

Cell Line	IC_50_ (μΜ)
HL-60	2.3 ± 1.0
CCRF/CEM	2.4 ± 1.0
LoVo	2.5 ± 1.6
Jurkat	3.4 ± 1.7
Daudi	3.8 ± 1.9
KHOS	5.0 ± 2.0
MDA-MB-231-TXSA	8.0 ± 3.5
STSA	15.4 ± 5.2
MCF-12 F	15.5 ± 5.5
MCF-7	16.0 ± 3.5
Raji	16.3 ± 6.1
A549	19.1 ± 10
Ishikawa	21.1 ± 10
PC-3	33.6 ± 9.7
PBMCs	>60

Cells were incubated with increasing concentrations of KC-53 (0–60 μΜ) for 48 h. The IC_50_ values were calculated from MTT viability curves. The data are expressed as the mean ± SD of three independent experiments performed in triplicate

**Fig. 2:**
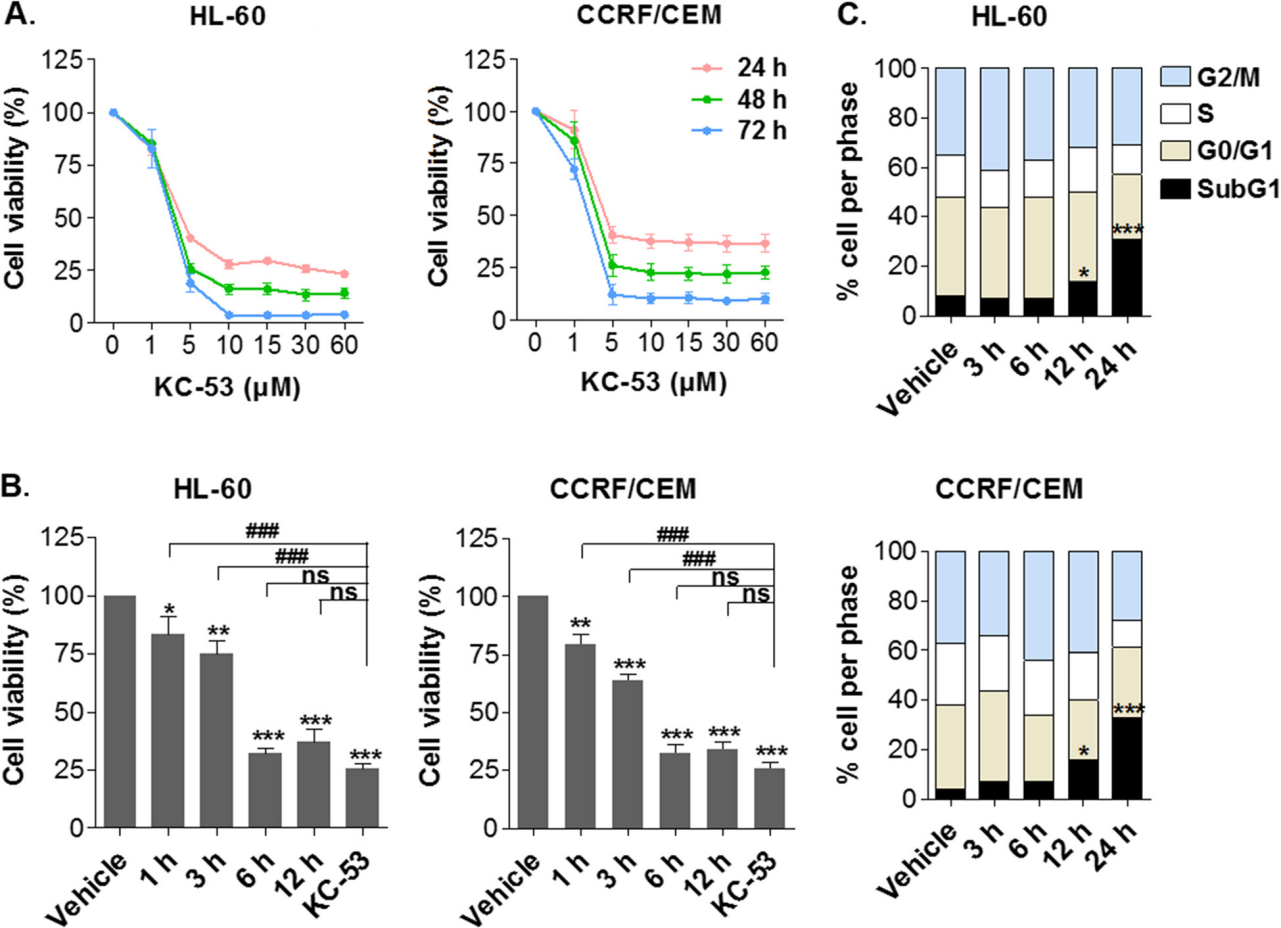
Effects of KC-53 on leukemic cellular proliferation and cell cycle progression. **a** Cells were treated with increasing concentrations of KC-53 for the times indicated. Cell viability determined by the MTT assay, is expressed as percentage of survival in comparison to vehicle treated controls. **b** Cells were exposed to 0 or 5 μΜ of KC-53 followed by removal of the agent after 1, 3, 6 or 12 h incubation and cell viability was determined after 48 h recovery in drug-free medium. A positive control, where KC-53 was not removed, is shown for comparison. **c** Cells were treated with 0 or 5 μM KC-53 for the times indicated and cell cycle was assessed by FACS analysis. The results represent the mean ± SEM of three replicates and are representative of three different experiments. (*p value <0.05, **p value <0.01, ***p value <0.001, ###*p* value <0.001)

KC-53 was found to reduce HL-60 and CCRF/CEM cell growth in a dose- and time- depended manner producing maximum reduction in cell viability at 10 μM in HL-60 and at 5 μM in CCRF/CEM (Fig. 2a). It became apparent from these growth response curves that the effect of the agent was almost immediate. To follow-up on this observation, we examined the possibility that the effects of KC-53 were irreversible. Towards this objective, we exposed the cells to KC-53 for 1, 3, 6 and 12 h followed by a post-treatment recovery period in agent-free medium for 48 h. In both cell lines viability was only partially restored when KC-53 was removed after 1 or 3 h of treatment (Fig. 2b). Treatments for 6 and 12 h produced a similar effect to that of continuous exposure. Thus, after 6 h of treatment the compound produced an irreversible inhibition of cell growth. The observed growth inhibitory effect of KC-53 in leukemic cells was not accompanied by any significant changes in the distribution of cell cycle phases as determined by flow cytometry (Fig. 2c). However, after 12 and 24 h of treatment there was an increase in Sub-G1 phase, indicative of apoptosis.

### KC-53 induces apoptosis in HL-60 and CCRF/CEM cells

**Fig. 3:**
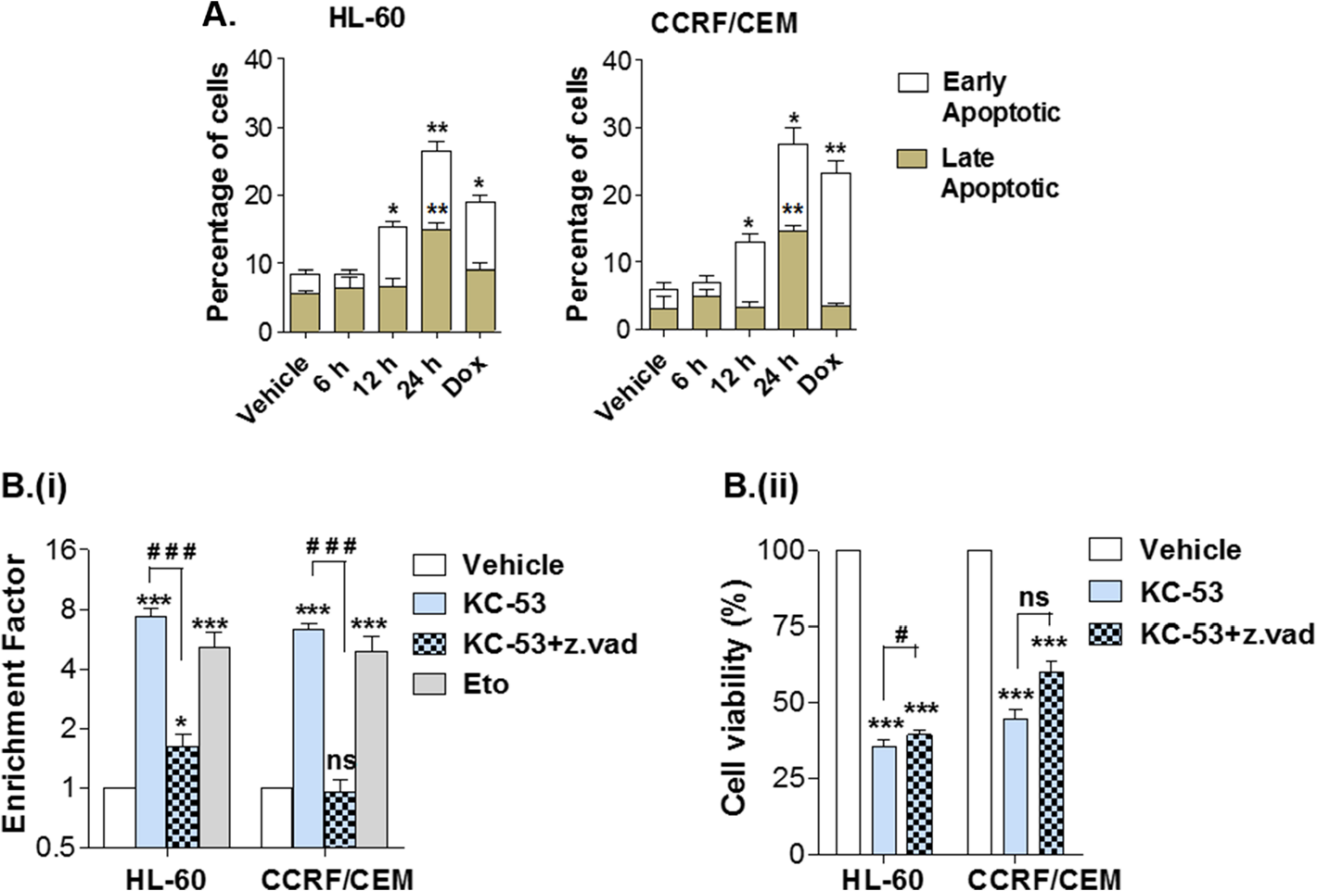
Effects of KC-53 on cell apoptosis and DNA integrity. **a** Cells were treated with 0 or 5 μΜ KC-53 for the indicated time points and apoptosis was assessed with Annexin-V/PI staining. Statistical significance was determined by comparing treated samples with the corresponding population of the vehicle control. For comparison, Doxorubicin (Dox) at 0.5 μΜ was used as positive control. **b** (**i**) Cells were treated with 0 or 5 μΜ KC-53 in the presence or absence of 20 μM z.vad.fmk for 24 h. The presence of nucleosomes in the cytoplasm was determined with the ELISA cell death detection kit and is expressed as Enrichment Factor. Etoposide (Eto) at 5 μΜ was used as positive control. (**ii**) Cells were treated with vehicle control or 5 μΜ KC-53 in the presence or absence of 20 μM z.vad.fmk, as shown, for 24 h. Cell viability was assessed with the MTT assay. The results represent the mean ± SEM of two replicates and are representative of three independent experiments. (*p value <0.05, **p value <0.01, ***p value <0.001, #*p* value <0.05, ###*p* value <0.001)

The possible induction of apoptosis by KC-53 was initially evaluated with the use of Annexin-V-FITC/PI assay. As indicated in Fig. 3a, within 12 h of treatment there was a significant increase in the early apoptotic fraction of both cell lines. After 24 h of treatment, 26.5 % of HL-60 and 27.5 % of CCRF/CEM cells were characterized as early apoptotic whereas, 15 % of cells from both cell lines were in the late apoptotic stage.

Apoptotic induction was further analyzed by the ELISA cell death kit which enables the detection of mono- and oligo-released nucleosomes in the cytosol. KC-53 induced substantial DNA fragmentation in HL-60 cells (7.4 fold increase compared to the control) and in CCRF/CEM cells (6.4 fold increase compared to the control) (Fig. 3b (i)). In the presence of the pan caspase inhibitor, z.vad.fmk, DNA fragmentation was significantly reduced in HL-60 cells and it was fully abolished in CCRF/CEM cells. These data suggest that activation of caspase cascades is predominantly involved in KC-53-induced apoptosis. DNA analysis was also performed with the Comet Assay where KC-53-induced DNA damage was evident within 9 h of treatment (Additional file 3) and was exclusively attributed to apoptosis as no cellular ROS production was detected (Additional file 4).

Even though co-incubation of KC-53 with z.vad.fmk restored DNA fragmentation, it did not fully restore the viability of cells (Fig. 3b (ii)). HL-60 viability increased from 43 to 60 % in the presence of z.vad.fmk while no alterations were observed in the viability of CCRF/CEM. These findings indicate that inhibition of cell proliferation by KC-53 might be mediated by both caspase -dependent (CD) and -independent (CID) programmed cell death in a cell-context-specific manner.

### KC-53 promotes apoptosis through activation of the TNFR1 signaling pathway

**Fig. 4:**
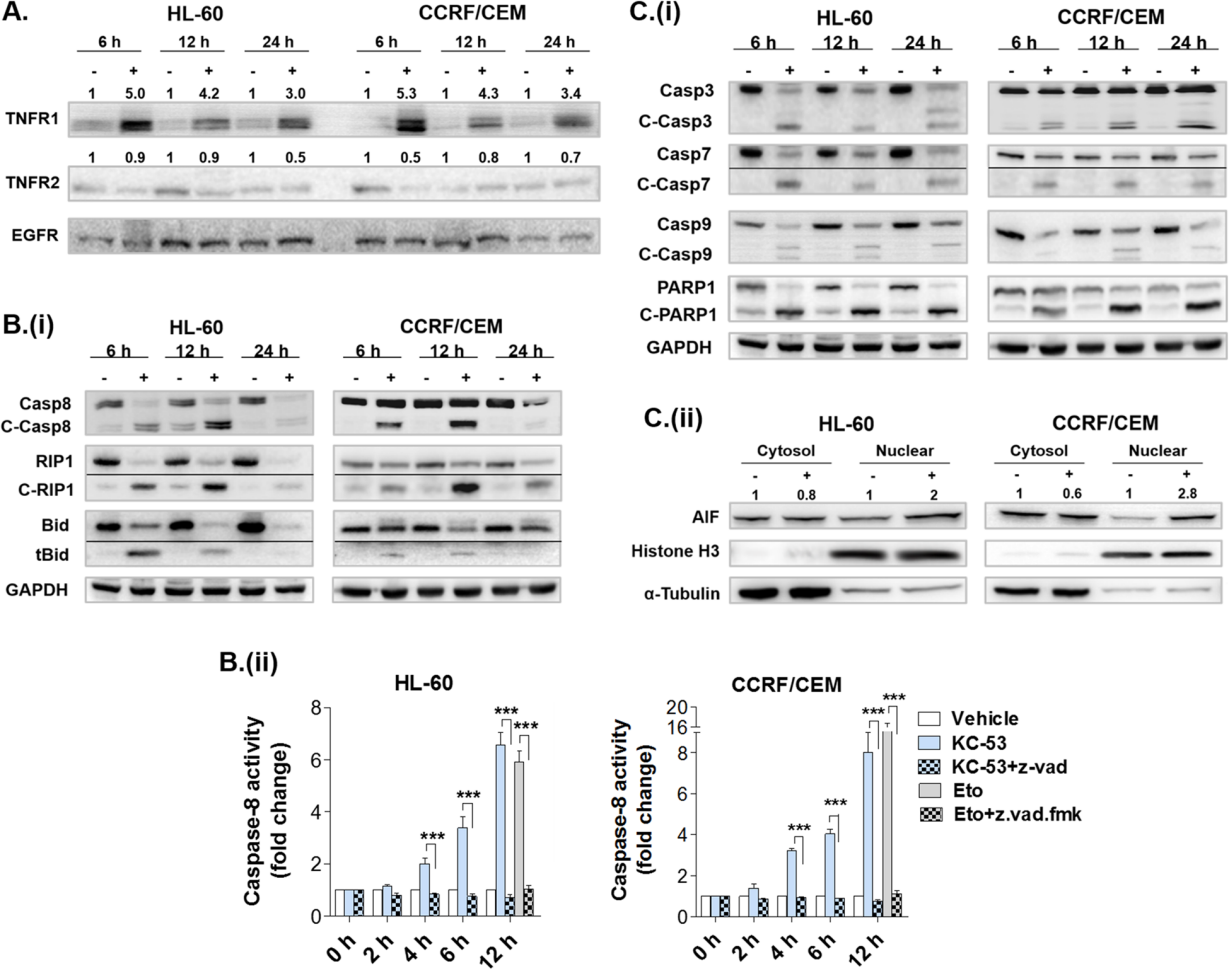
Effects of KC-53 on Caspases and regulatory molecules of the apoptotic pathways in leukemia cells. **a** Cells were treated with 0 or 5 μΜ KC-53 for the indicated time points. TNFRs membrane expression levels were analyzed by immunoblotting. EGFR was used as a loading control and the TNFRs levels were quantified and normalized in comparison to the EGFR levels. **b** (**i**) Cells were treated with 0 or 5 μΜ KC-53 for the indicated time points. (**ii**) Cells were treated with 0 or 5 μΜ KC-53 in the presence or absence of 20 μM z.vad.fmk. Caspase-8 enzymatic activity was determined as described in Methods. Etoposide (Eto) added at 5 μΜ was used as a positive control. **c** (**i**) Cells were treated with 0 or 5 μΜ KC-53 for the indicated time points followed by immunoblotting. (**ii**) Cells were incubated with 0 or 5 μΜ KC-53 for 6 h and cytosolic and nuclear protein extracts were prepared. The numbers on top of each band represent intensity values and are expressed as fold change in comparison to the control. The results in panels **a**, **b** (**i**) and **c** are representative of three repetitions. The results in **b** (**ii**) panels represent the mean ± SEM of two replicates and are representative of three independent experiments. (***p value <0.001)

To fully characterize the apoptotic pathway being induced by KC-53, we monitored the potential activation of caspases and any changes in the membrane death receptor levels. In both HL-60 and CCRF/CEM cell lines an increase in membrane-associated TNFR1 was evident with KC-53 treatment for 6 up to 24 h (Fig. 4a). The corresponding protein levels of TNFR2 were not significantly affected by the treatment (Fig. 4a). The levels of death receptors FAS, DR3 and DR5, decoy receptor DcR3 as well as those of adaptor proteins FADD and TRADD remained relatively unaffected (Additional file 5).

The increase in TNFR1 levels was accompanied by strong activation and detection of the cleaved 43/41 kDa forms of Caspase-8 (C-Casp8) and proteolytic inactivation of RIP1 (C-RIP1) (Fig. 4b (i)) indicating that the extrinsic pathway of apoptosis is triggered. The crosstalk between the extrinsic and the intrinsic pathways is well established and occurs though Caspase-8 cleavage and activation of the pro-apoptotic protein Bid [27, 28]. To investigate this scenario we determined the expression levels of cleaved/truncated Bid (tBid). KC-53 administration resulted in the detection of the 15 kDa tBid fragment in both cell lines (Fig. 4b (i)). The cleavage of Bid occurred in the early stage of apoptosis (6 h) parallel with Caspase-8 activation. In both cell lines the amount of the 15 kDa peptide was constant during the time course of apoptosis and became undetectable after 24 h, possibly due to further degradation.

Caspase-8 enzymatic activity was verified with the use of fluorometric protease assay kit, in the presence or absence of z.vad.fmk. KC-53 produced a significant increase of Caspase-8 activity within 4 h of treatment. Specifically, after 12 h there was a 6.6 fold increase of Caspase-8 activity in HL-60 cells and an 8 fold increase in CCRF/CEM cells (Fig. 4b (ii)). The effects of KC-53 on Caspase-8 activity were fully reversed by z.vad.fmk in both cell lines.

To further evaluate the apoptotic effect of KC-53, we monitored the expression of the executor Caspases, −3 and −7 and their substrate PARP1. KC-53 markedly increased the active, cleaved forms of Caspases −3 (C-Casp3; 19/17 kDa) and −7 (C-Casp7; 20 kDa), which was evident within 6 h of treatment and persisted for 24 h post-treatment (Fig. 4c (i)). Caspase activation was accompanied by a decrease in the levels of the full length 116 kDa PARP1 and appearance of the cleaved 89 kDa form (C-PARP1) (Fig. 4c (i)). Similarly, KC-53 induced activation of the initiator Caspase-9 (C-Casp9; 37/35 kDa) (Fig. 4c (i)) and translocation of apoptosis inducing factor, AIF from the cytosol to the nucleus (Fig. 4c (ii)). In HL-60 cells, the nuclear levels of AIF increased up to two fold and in CCRF/CEM up to 2.8 fold following 6 h of treatment (Fig. 4c (ii)). Both Caspase-9 activation and AIF release are characteristics of mitochondrial outer membrane permeabilization (MOMP) apparently induced by tBid. These findings support the involvement of mitochondrial-mediated intrinsic pathway in the induction of apoptosis by KC-53. Furthermore, as AIF is a mediator of CID apoptotic mechanisms, these data provide further support to our previous observation (Fig. 3b (ii)) regarding the possible involvement of CID mechanisms by KC-53 action.

### KC-53 inhibits the activation of IκΒα and the translocation of p65/NF-κΒ to the nucleus

TRAF2 is required for the assembly of kinases regulating the phosphorylation and degradation of the NF-κΒ inhibitor, IκBα. We therefore investigated whether KC-53 treatment can affect the downstream molecular events of the TNFR1/NF-κΒ signalling as well as NF-κΒ translocation to the nucleus. This was evaluated by monitoring the phosphorylation status and protein levels of TRAF2 and IκBα following induction by TNFα in the absence or presence of KC-53.

**Fig. 5:**
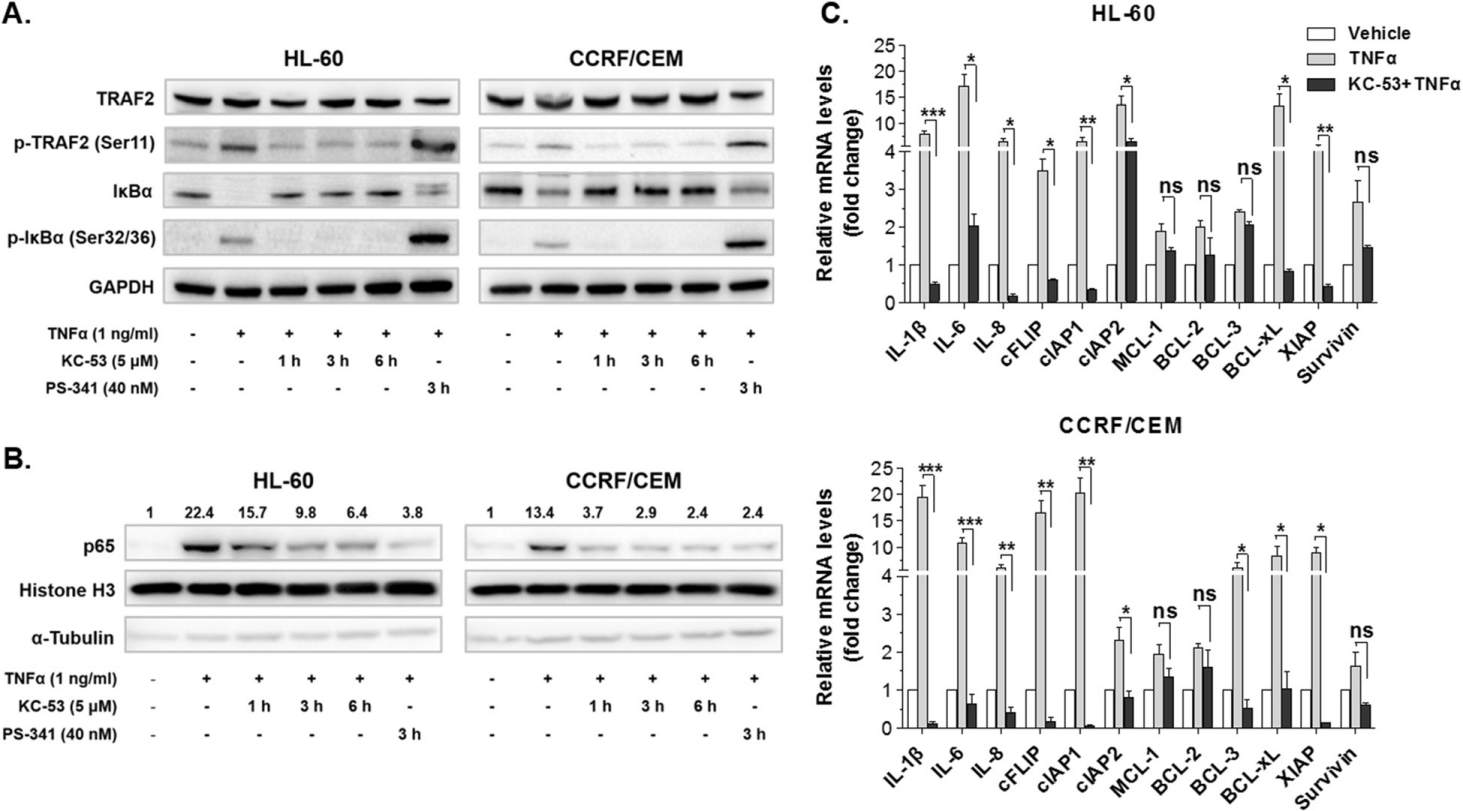
Effects of KC-53 on TRAF2 and IκΒα phosphorylation, and p65 translocation to the nucleus. **a** Cells were incubated with vehicle, KC-53 or PS-341 (for the times indicated) followed by incubation with TNFα for 20 min. Whole cell extracts were analyzed by immunoblotting. **b** Cells were incubated to either vehicle, KC-53 or PS-34 as indicated followed by the addition of TNFα for 20 min. The nuclear localization of p65 was examined by immunoblotting. Histone H3 levels were determined as a loading control and the α-Tubulin levels as an indicator of cytosolic contamination. Numbers signifies intensity values and are expressed as fold change compared to the control. **c** Cells were subjected to treatments either with the vehicle or TNFα (1 ng/ml) for 4 h, or KC-53 (5 μΜ) for 6 h plus TNFα for 4 h. The PCR products were normalized to those obtained from GAPDH mRNA amplification. The results in panels (**a**) and (**b**) are representative of three repetitions. The results in (**c**) panels represent the mean ± SEM of two replicates and are representative of three independent experiments. (*p value <0.05, **p value <0.01, ***p value <0.001)

We found that TNFα increased the phosphorylation levels of TRAF2 (Ser11) by 3.5 fold in HL-60 cells and by 2.2 fold in CCRF/CEM cells (compared to the control levels) while KC-53 fully attenuated these effects (Fig. 5a). Furthermore, in both cell lines, pretreatment with KC-53 fully abolished the TNFα-induced phosphorylation of IκBα on Ser32/36 without affecting the overall IκBα levels (Fig. 5a). Nuclear extraction and immunoblotting against the p65 subunit of NF-κΒ showed a time-depended decrease in the TNFα-induced nuclear translocation of p65 in response to KC-53 in both cell lines (Fig. 5b). An impressive 71 % and 82 % decrease in the p65 nuclear levels in HL-60 and CCRF/CEM cells was noted respectively, after 6 h of treatment, apparently due to decreased translocation. The above findings, clearly show that KC-53 stabilizes the p65/IκBα complex by inhibiting TNFα-induced phosphorylation on TRAF2 and IκBα, preventing in this manner p65 translocation to the nucleus.

The efficiency of KC-53 in inhibiting TRAF2 and IκBα phosphorylation and/or p65 translocation was also compared with the well-established proteasome inhibitor, Bortezomib (PS-341). As was expected, Bortezomib maintained the IκΒα levels without abolishing the phosphorylation on Ser32/36 neither that of TRAF2 on Ser11 (Fig. 5a). Nonetheless Bortezomib reduced p65 nuclear levels by 83 % compared to the TNFα-treated samples (Fig. 5b). Thus, the effects of KC-53 on the nuclear levels of p65 are similar to those of Bortezomib although the mechanism by which this is achieved is different.

To further test the expectation that KC-53 hinders p65 transcriptional activity, we determined the mRNA levels of genes known to be transcriptionally activated by p65. We found that KC-53 robustly inhibited the TNFα-induced transcription of the pro-inflammatory cytokines; *IL -1β, −6 and −8* and the pro-survival mediators; *c-FLIP, cIAP-1, cIAP-2, BCL-xL* and *XIAP* whereas *BCL-2, MCL-1* and *Survivin* levels were not significantly affected (Fig. 5c). These results are consistent with the conclusion that KC-53 shifts the balance between the TNFR1-mediated pro-survival and pro-apoptotic signals in favour of the latter and thus, inhibits the activation of the NF-κΒ in HL-60 and CCRF/CEM cells. Therefore, the antiproliferative activity of KC-53 in leukemia cells might be also attributed in the inhibition of the NF-κΒ survival axis.

### Silencing of FADD protects leukemic cells from KC-53 apoptotic effects

**Fig. 6:**
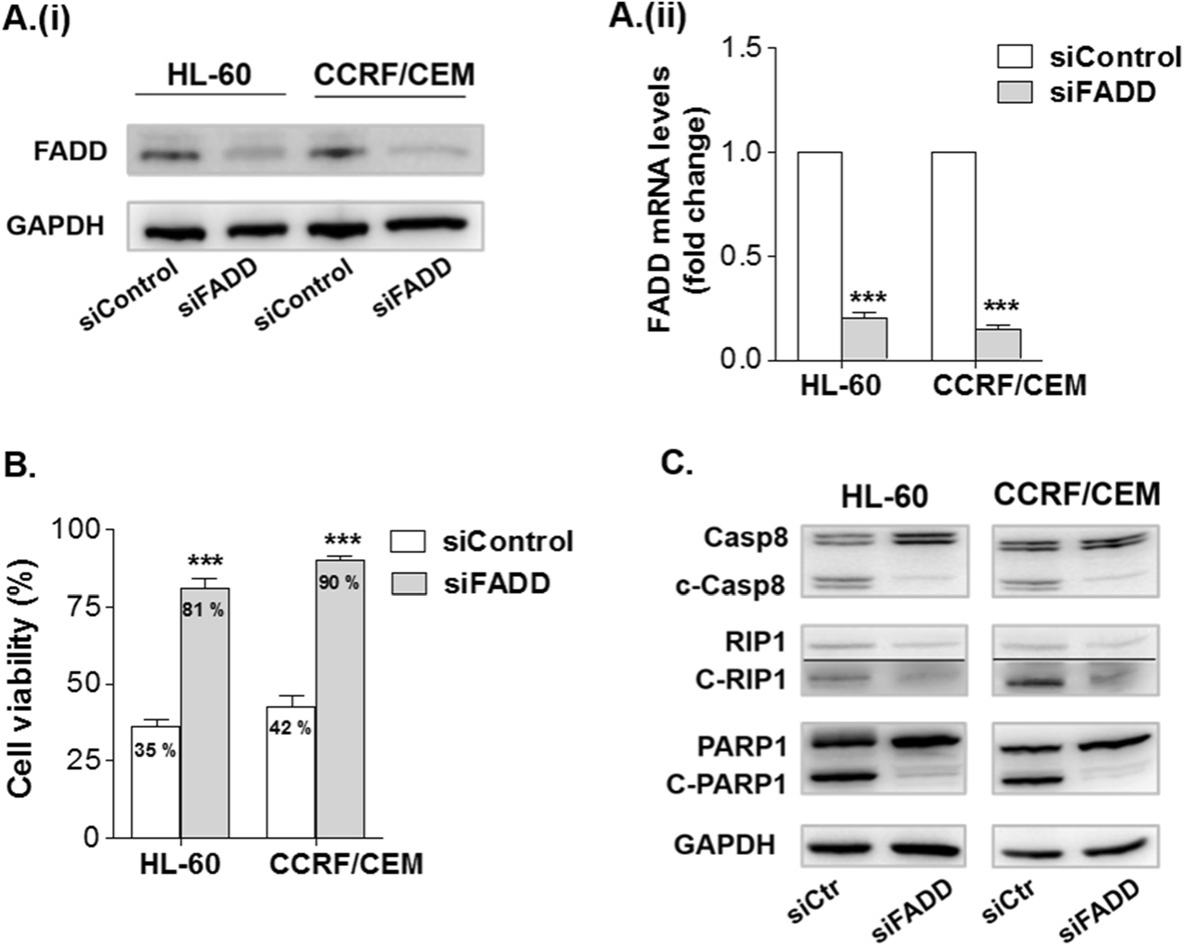
Effects of FADD silencing on the antiproliferative and apoptotic properties of KC-53 in leukemic cells. **a** HL-60 and CCRF/CEM cells were transiently transfected with siRNA control or siRNA FADD followed by (**i**) immunoblotting for the detection of FADD protein levels and (**ii**) qPCR for measuring FADD mRNA levels. **b** Transfected cells were treated with 0 or 5 μΜ KC-53 for 24 h and cell viability was determined with the MTT assay. **c** Transfected cells were treated with 5 μΜ KC-53 for 6 h and protein levels were determined by immunoblotting. The results in panels **a** (**i**) and **c** are representative of three repetitions. The results in **a** (**ii**) and **b** panels represent the mean ± SEM of three replicates and are representative of three separate experiments. (***p value <0.001)

In order to investigate whether KC-53 apoptotic effects are directly linked to TNFR1 pro-apoptotic signaling axis we used siRNA for inhibiting the expression of FADD (Fig. 6a). It is known from previous reports that, FADD is a core protein of the pro-apoptotic complex facilitating Caspase-8 activation soon after TNFR1 activation thus, promoting apoptosis [29]. FADD silencing significantly restored cell viability by up to 78 and 90 % in HL-60 and CCRF/CEM respectively (Fig. 6b). These data suggest that downregulation of FADD confers resistance to KC-53 and that, the cytotoxic effects of the agent may be mediated through the formation of the pro-apoptotic complex. In agreement with this, in the absence of FADD, KC-53 was not able to activate Caspase-8, nor to promote the proteolytic inactivation of RIP1 or PARP1 (Fig. 6c). Taken together, the results presented here support that the FADD/Caspase-8/RIP1 signaling axis plays a crucial role in KC-53 induced apoptosis of HL-60 and CCRF/CEM cells.

## Discussion

**Fig. 7:**
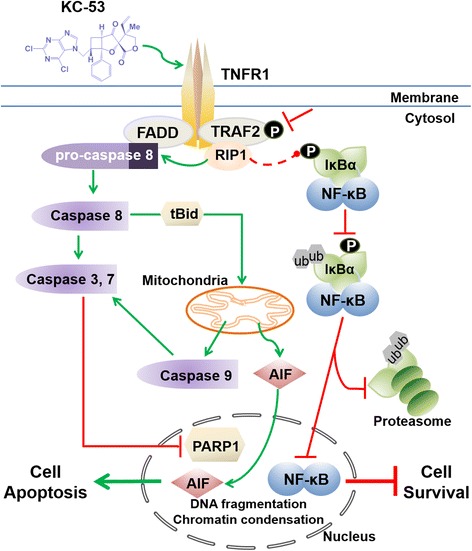
Sequence of molecular events leading to anti-proliferative and pro-apoptotic effects of KC-53 in leukemic cells. KC-53 stimulates TNFR1 and inhibits TRAF2 phosphorylation. RIP1 dissociates from TRAF2 and binds to the FADD/pro-caspase 8 complex. This leads to the activation of the procaspase-8 which in turns cleaves and inactivates RIP1. Caspase-8 triggers Bid cleavage, activation of effectors Caspases, −3 and −7 and inactivation of PARP1 promoting cell apoptosis. tBid leads to Caspase-9 activation and AIF release and translocation to the nucleus. The absence of RIP1 from TRADD/TRAF2 complex diminishes the phosphorylation of IκΒα by downstream kinases. As a result, IκΒα is not phosphorylated and fails to be ubiquitinated and degraded by proteasome. Subsequently, NF-κB remains in complex with IκΒα, fails to translocate to the nucleus and cell survival signaling is hindered. *P* phosphorylation, *ub* ubiquitination

Although the overall survival rate of leukemia patients has dramatically increased in the past decade, there is still a strong need for discovering new therapeutic agents with higher specificity and milder side-effects. In the present study we evaluated for the first time the anticancer efficacy of KC-53. Importantly, we discovered that, KC-53 reduces cancer cell viability in a dose-depended manner in all cell lines and exhibits the highest cytotoxicity towards HL-60 (AML/APL) and CCR/CEM (ALL) leukemic cell lines. Remarkably, the normal PBMCs were relatively resistant to the anti-proliferative effects of the agent, suggesting that the KC-53 inhibitory effects are selective against cancer cells. We show that KC-53 efficiently and irreversible inhibits cells growth, promoting rabidly CD and CID apoptotic cell death. The molecular events leading to reduced survival and apoptosis have been methodically unravelled in this study and are illustrated in Fig. 7.

Initially, the agent up-regulates membrane-bound TNFR1 followed by activation of Caspase-8, RIP1 proteolysis and activation of Caspases, −3, −7 and −9. The failure of restoring cell viability in the presence of the pan-caspase inhibitor, z.vad.fmk suggests that CID mechanisms may also be involved in the mode of action of KC-53 (Fig. 3b). This prediction is supported by the release and translocation of AIF from the mitochondrial to the nucleus (Fig. 4c (ii)) which is commonly induced by Calpains and Cathepsins [30]. Interestingly, it has been previously shown that when Caspase-8 activity is blocked, the cell uses necroptosis as an alternative cell death pathway (Reviewed in [31]). RIP1 and RIP3 kinase activities are crucial for this alternative CID pathway induced by death receptors, including TNFR1. Thus, activation of necroptosis by KC-53 cannot be excluded at this point.

The participation of FADD in a complex with Caspase-8 and RIP1 is known to be required for the activation of the extrinsic pathway through TNFR1 [32]. Down regulation of FADD with siRNA inhibited the formation of the pro-apoptotic complex and, consequently, the FADD-deficient HL-60 and CCRF/CEM cells developed resistance to the anti-proliferative and apoptotic effects of KC-53. These results strongly suggest that, following TNFR1 activation, one of the primary apoptotic effects of KC-53 is the formation of the FADD/Caspsase-8/RIP1 pro-apoptotic complex. Importantly, our data clearly show that KC-53 concurrently inhibits pro-survival NF-κΒ signaling. We determined that KC-53 strongly inhibited the TNFα-induced phosphorylation on Ser32/36 of IκΒα. The hypo-phosphorylated form of IκΒα stabilizes the cytoplasmic IκΒα/p65 complex, blocking in this manner p65 translocation to the nucleus. Consequently, KC-53 TNFα-stimulated gene expression of both pro-survival and pro-inflammatory p65-mediators. This effect of KC-53 could also explain the previously reported anti-inflammatory activity of KC-53 [26].

The use of anti-TNF antibodies and specific agents to block TNFRs and NF-κB activation has been a valuable approach against inflammatory diseases [33]. Proteasome inhibitors [34] and IKK inhibitors [35, 36] have also been used to block the NF-κB pathway and to enhance the sensitivity of cancer cells to apoptosis. For instance, the proteasome inhibitor Bortezomib is currently approved for the treatment of mantle cell lymphoma [37, 38]. However, due to low specificity for cancer cells versus normal cells, it causes severe side effects [37]. The proteasome, which is responsible for IκΒα degradation has many other vital cellular functions and it may also not be feasible to block it for prolonged periods. Consequently, hindering NF-κB by controlling upstream regulatory molecules such as RIP1 and TRAF2 might be a more efficient and less cytotoxic approach in comparison to Bortezomib for the treatment of blood diseases. Preclinical evidence for the importance of TRAF2 and RIP1 as targets for anticancer drugs is based on two observations: (i) that inactivating mutations of TRAF2 is a dominant-negative event, neutralizing TNFα-induced NF-κB activation [6, 39] and, (ii) RIP1-null cells or mice do not undergo TNFα-induced cell death (Reviewed in [40, 41]). Our data revealed that, KC-53 induced a robust degradation of RIP1 and dramatically inhibited the TNFα-induced phosphorylation on Ser11 of TRAF2. As such, the absence of RIP1 and phospho-TRAF2, from the pro-survival complex blocked the downstream phosphorylation events leading to NF-κΒ (space) activation.

Unlike most chemotherapeutic drugs, ligands of the TNF family induce apoptosis in a p53-independent manner and are promising alternatives to conventional chemotherapy. Specifically for leukemia, mutational inactivation of the *p53* gene which mainly regulates apoptosis via the DNA damage-induced intrinsic pathway, reduces cancer cell sensitivity to conventional treatments [12, 13]. In this aspect, KC-53 enables the crosstalk between the extrinsic and intrinsic pathway enabling cell lines with non-functional p53 to bypass the p53-mitochondrial block. Caspase-8-mediated cleavage of Bid provides the link between death receptor stimulation and mitochondrial apoptotic events. In both HL-60 (*p53*−/−) and CCRF/CEM (*p53*mut) KC-53 promoted the activation of Bid and the cleavage of Caspase-9. tBid has the ability to accumulate at mitochondria and to initiate MOMP [27]. MOMP in turn results in the release of pro-apoptotic factors from the mitochondrial intermembrane space, including cytochrome *c*, triggering formation of the apoptosome and activation of Caspase-9. Collectively, our data identified the key role of the TNFR1 pathway in KC-53-induced apoptosis, where the engagement of the mitochondrial system amplified cell death. This may have clinical implications, since it may critically reduce the time required for execution of the death program. This also suggests that KC-53 may find applications in the treatment of p53 mutant cancers and help to overcome resistance.

The idea to specifically target the extrinsic pathway to trigger apoptosis in malignant cells is attractive for cancer therapy since death receptors have a direct link to the death machinery. However, the clinical application of TNFα and Fas is hampered by severe toxic side effects [42, 43]. TRAIL remains promising as a cancer therapeutic, despite the fact that many tumors remain refractory towards treatment with TRAIL [44–46]. In most, if not all, clinical studies the lack of efficacy was probably attributed to their inability to overcome the mitochondrial block [47–49]. KC-53 appears to be a strong candidate for TNFR1 activation and may help to overcome TRAIL resistance and/or increase malignant cell sensitivity to chemotherapy. Our work also represents a new concept in the design of TNFR1-targeted therapies as this is the first time that an agent has been reported to stimulate efficiently TNFR1 inhibiting cancer cell growth and concurrently eliminate the activation of NF-κB.

## Conclusions

Our findings show for the first time that, KC-53 effectively triggers apoptosis by facilitating both the extrinsic and intrinsic pathway, bypassing the p53-mitochondrial block and hindering the p65/NF-κB survival cascade in APL and ALL cells. Because of these qualities we anticipate that KC-53 is very likely to find applications, either as a single agent, or in combination with other conventional chemotherapeutic agents in targeted therapeutics against acute leukemias.

## Additional files

Additional file 1: Nucleotide sequences of PCR primers. (PDF 186 kb) Additional file 2: The effect of KC-53 on the survival of various human cancer cell lines, on PBMCs and on the immortalized “normal” cell line, MCF-12 F. Cells were exposed to increasing concentrations (0–60 μΜ) of KC-53 for 48 h. Cell survival was determined with the MTT cell viability assay and is expressed as percentage of survival compared to vehicle controls. The results represent the mean ± SEM of three replicates and are representative of at least three different experiments. (TIF 54 kb) Additional file 3: KC-53 induces DNA damage in HL-60 and CCRF/CEM cells. HL-60 and CCRF/CEM cells were treated with vehicle control or 5 μM of KC-53 for the times indicated and DNA damage was evaluated with the Comet assay. For comparison, cell samples were treated with 100 μΜ Η_2_Ο_2_ for 30 min which is known to produces oxidative DNA damage (positive control). (**i**) Images were obtained by fluorescence microscopy and show comet fields after SYBR Green I staining. (**ii**) The DNA damage was quantified based on the comet tail length. The results are representative of three independent experiments. (**p* value <0.05, ****p* value <0.001). (TIF 522 kb) Additional file 4: KC-53 does not promote the generation of reactive oxygen species in leukemic cells. HL-60 and CCRF/CEM cells were treated with vehicle control or 5 μM of KC-53 in the presence or absence of 1 mM Sodium pyruvate (SP) for the indicated time points. Cells were also treated with 100 μΜ Hydrogen peroxide (Η_2_Ο_2_) in the presence or absence of 1 mM SP as controls. ROS production was determined with the DCFH-DA assay. The treatments were performed in duplicate and represent the mean ± SEM of three independent experiments. (TIF 55 kb) Additional file 5: The expression of death receptors and adaptor proteins upon KC-53 administration. HL-60 and CCRF/CEM cells were treated with 0 or 5 μΜ KC-53 for the indicated times prior protein extraction. Proteins were separated by SDS-PAGE and immunoblotted with the indicated antibodies. The results are representative of three repetitions. (TIF 335 kb)

## Abbreviations

ALL: acute lymphocytic leukemia
AML: acute myelocytic leukemia
APL: acute promyelocytic leukemia
CD: caspase dependent
CID: caspase independent
FADD: fas-associated death domain
IKK: IκΒ kinase
IκΒα: inhibitory protein of NF-κΒ
NF-κB: nuclear factor κΒ
PBMCs: peripheral blood mononuclear cells
rhTNFα: recombinant human TNFα
RIP1: receptor-interacting protein 1
TNFR1: tumor necrosis factor receptor 1
TNFα: tumor necrosis factor alpha
TRAF2: TNF receptor-associated factor 2
