# Supplementary material for: Selective activation of TNFR1 and NF-κB inhibition by a novel biyouyanagin analogue promotes apoptosis in acute leukemia cells
Source: BMC Cancer. 2016 Apr 20;16:279. doi: 10.1186/s12885-016-2310-5 (PMC4839067; doi:10.1186/s12885-016-2310-5)
Supplement: Additional file 1: — Nucleotide sequences of PCR primers. (PDF 186 kb) [file 12885_2016_2310_MOESM1_ESM.pdf]

Gene	Forward Primer	Reverse Primer
**IL-1β**	$^{5'}$TCCAGCTACGAATCTCCGAC$_{3'}$	$^{5'}$ACCAGCATCTTCCTCAGCTT$_{3'}$
**IL-6**	$^{5'}$AGTCCTGATCCAGTTCCTGC$_{3'}$	$^{5'}$CATTTGTGGTTGGGTCAGGG$_{3'}$
**IL-8**	$^{5'}$CAGTTTTGCCAAGGAGTGCT$_{3'}$	$^{5'}$TTGGGGTGGAAAGGTTTGGA$_{3'}$
**BCL-xL**	$^{5'}$TAAACTGGGGTCGCATTGT$_{3'}$	$^{5'}$TGGATCCAAGGCTCTAGGTG$_{3'}$
**BCL-2**	$^{5'}$CAGCCCAGACTCACATCACC$_{3'}$	$^{5'}$CATGTGTGTGGAGAGCGTCA$_{3'}$
**BCL-3**	$^{5'}$AAGAAACCGTGCAGCTCTTG$_{3'}$	$^{5'}$CCGCTCTTAATGTCCACTGC$_{3'}$
**XIAP**	$^{5'}$TGGGGTTCAGTTTCAAGGAC$_{3'}$	$^{5'}$TGCAACCAGAACCTCAAGTG$_{3'}$
**cIAP1**	$^{5'}$GCATTTTCCCAACTGTCCAT$_{3'}$	$^{5'}$ATTCGAGCTGCATGTGTCTG$_{3'}$
**cIAP2**	$^{5'}$GCATTTTCCCAACTGTCCAT$_{3'}$	$^{5'}$ATTTTCCACCACAGGCAAAG$_{3'}$
**cFLIP**	$^{5'}$TGTGCCGGGATGTTGCTATA$_{3'}$	$^{5'}$CCGACAGACAGCTTACCTCT$_{3'}$
**MCL-1**	$^{5'}$ATGCCAAACCAGCTCCTACT$_{3'}$	$^{5'}$GCTGCATCGAACCATTAGCA$_{3'}$
**Survivin**	$^{5'}$GACGACCCCATAGAGGAACA$_{3'}$	$^{5'}$GACAGAAAGGAAAGCGCAAC$_{3'}$
**FADD**	$^{5'}$ACACAGAGAAGGAGAACGCA$_{3'}$	$^{5'}$GCCTGCTGAACCTCTTGTAC$_{3'}$
**GAPDH**	$^{5'}$CTGACTTCAACAGCGACACC$_{3'}$	$^{5'}$AAAGTGGTCGTTGAGGGCA$_{3'}$
