# Supplementary figures and images for: Selective activation of TNFR1 and NF-κB inhibition by a novel biyouyanagin analogue promotes apoptosis in acute leukemia cells
Source: BMC Cancer. 2016 Apr 20;16:279. doi: 10.1186/s12885-016-2310-5 (PMC4839067; doi:10.1186/s12885-016-2310-5)

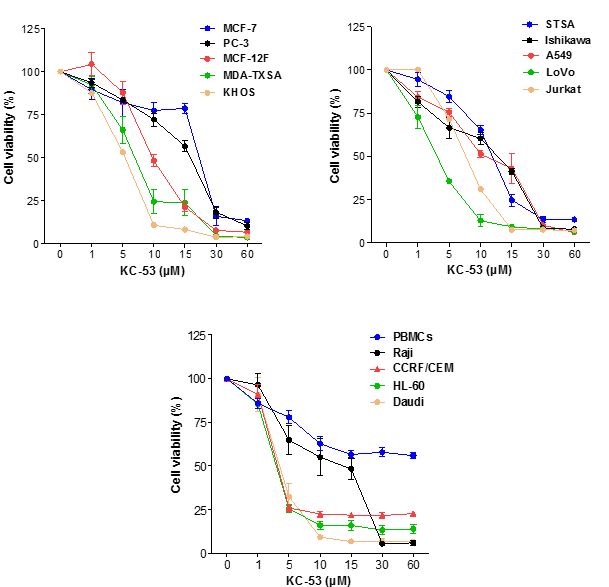

Supplement: Additional file 2: — The effect of KC-53 on the survival of various human cancer cell lines, on PBMCs and on the immortalized “normal” cell line, MCF-12 F. Cells were exposed to increasing concentrations (0–60 μΜ) of KC-53 for 48 h. Cell survival was determined with the MTT cell viability assay and is expressed as percentage of survival compared to vehicle controls. The results represent the mean ± SEM of three replicates and are representative of at least three different experiments. (TIF 54 kb) [file 12885_2016_2310_MOESM2_ESM.tif]

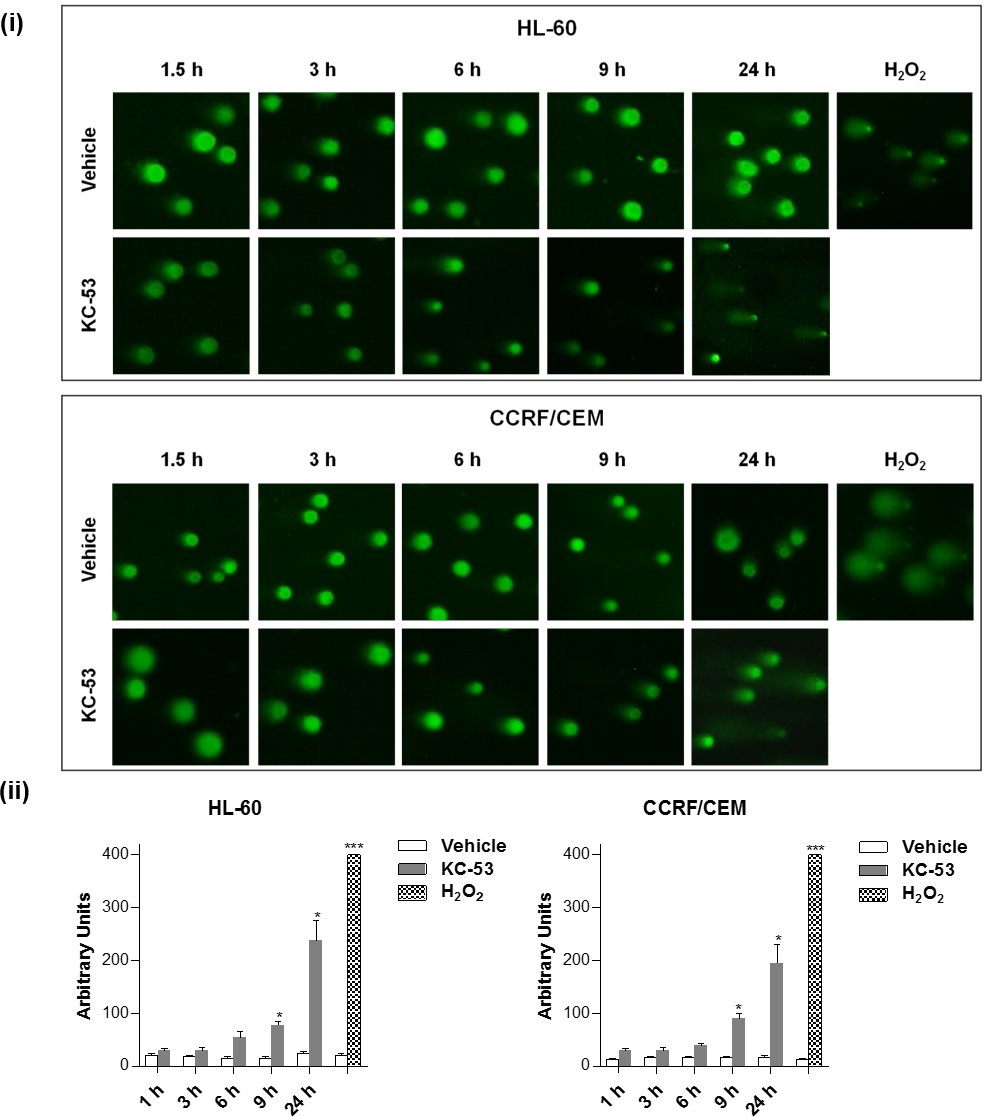

Supplement: Additional file 3: — KC-53 induces DNA damage in HL-60 and CCRF/CEM cells. HL-60 and CCRF/CEM cells were treated with vehicle control or 5 μM of KC-53 for the times indicated and DNA damage was evaluated with the Comet assay. For comparison, cell samples were treated with 100 μΜ Η2Ο2 for 30 min which is known to produces oxidative DNA damage (positive control). (i) Images were obtained by fluorescence microscopy and show comet fields after SYBR Green I staining. (ii) The DNA damage was quantified based on the comet tail length. The results are representative of three independent experiments. (*p value <0.05, ***p value <0.001). (TIF 522 kb) [file 12885_2016_2310_MOESM3_ESM.tif]

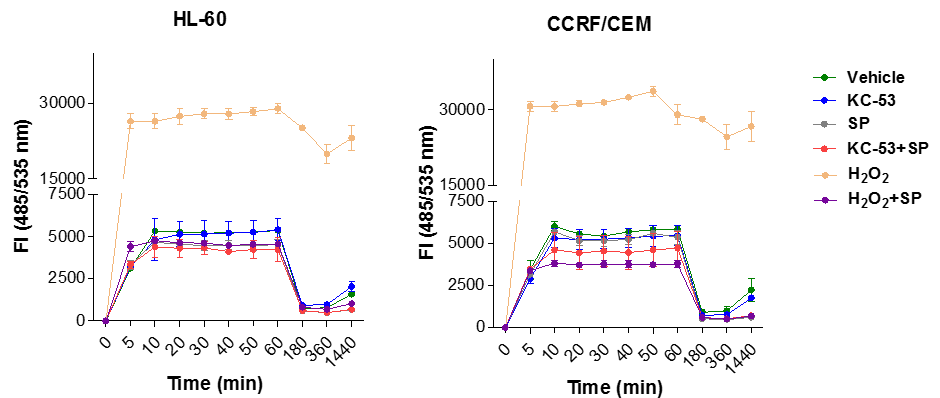

Supplement: Additional file 4: — KC-53 does not promote the generation of reactive oxygen species in leukemic cells. HL-60 and CCRF/CEM cells were treated with vehicle control or 5 μM of KC-53 in the presence or absence of 1 mM Sodium pyruvate (SP) for the indicated time points. Cells were also treated with 100 μΜ Hydrogen peroxide (Η2Ο2) in the presence or absence of 1 mM SP as controls. ROS production was determined with the DCFH-DA assay. The treatments were performed in duplicate and represent the mean ± SEM of three independent experiments. (TIF 55 kb) [file 12885_2016_2310_MOESM4_ESM.tif]

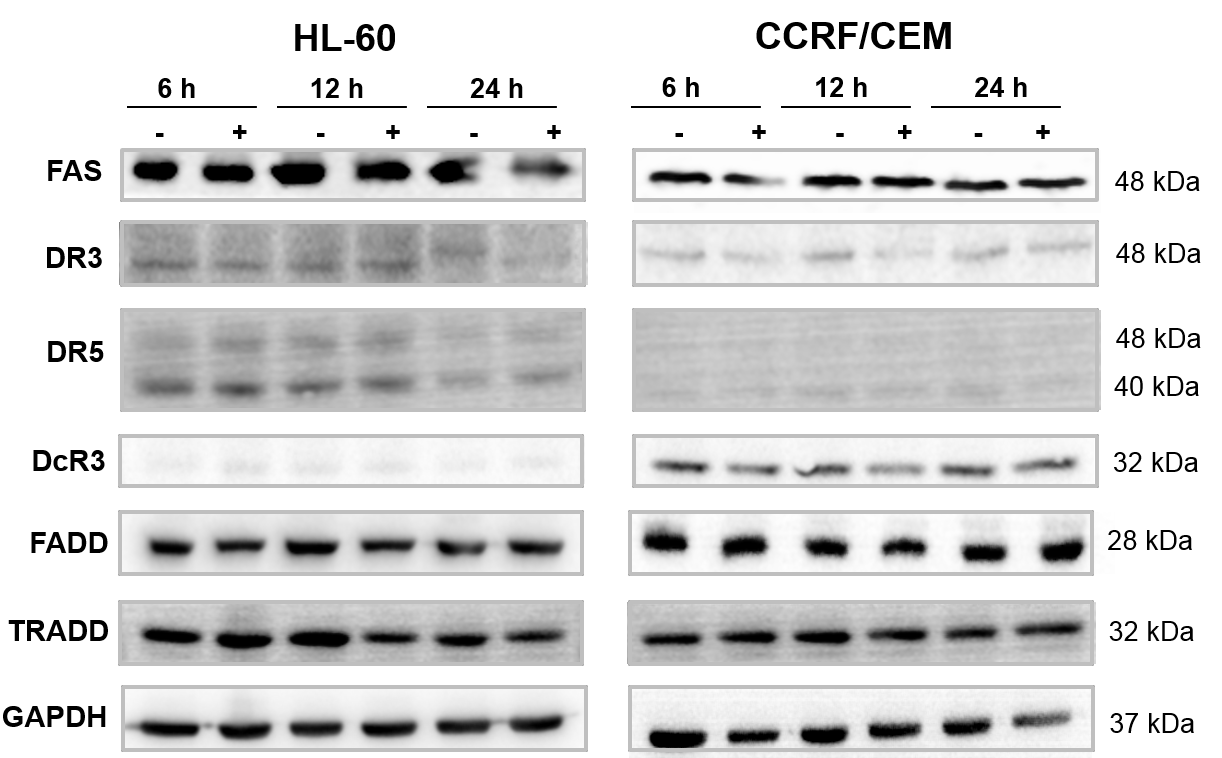

Supplement: Additional file 5: — The expression of death receptors and adaptor proteins upon KC-53 administration. HL-60 and CCRF/CEM cells were treated with 0 or 5 μΜ KC-53 for the indicated times prior protein extraction. Proteins were separated by SDS-PAGE and immunoblotted with the indicated antibodies. The results are representative of three repetitions. (TIF 335 kb) [file 12885_2016_2310_MOESM5_ESM.tif]
